# Development of sedentary behavior across childhood and adolescence: longitudinal analysis of the Gateshead Millennium Study

**DOI:** 10.1186/s12966-016-0413-7

**Published:** 2016-08-02

**Authors:** Xanne Janssen, Kay D. Mann, Laura Basterfield, Kathryn N. Parkinson, Mark S. Pearce, Jessica K. Reilly, Ashley J. Adamson, John J. Reilly

**Affiliations:** 1 University of Strathclyde, School of Psychological Science and Health, Glasgow, Scotland UK; 2 Institute of Health & Society, Newcastle University, Newcastle upon Tyne, England UK; 3 Human Nutrition Research Centre, Newcastle University, Newcastle upon Tyne, England UK

**Keywords:** Sitting, Accelerometry, Children, Adolescents, Longitudinal, Cohort

## Abstract

**Background:**

In many parts of the world policy and research interventions to modify sedentary behavior of children and adolescents are now being developed. However, the evidence to inform these interventions (e.g. how sedentary behavior changes across childhood and adolescence) is limited. This study aimed to assess longitudinal changes in sedentary behavior, and examine the degree of tracking of sedentary behavior from age 7y to 15y.

**Methods:**

Participants were part of the Gateshead Millennium Study cohort. Measures were made at age 7y (*n* = 507), 9y (*n* = 510), 12y (*n* = 425) and 15y (*n* = 310). Participants were asked to wear an ActiGraph GT1M and accelerometer epochs were defined as sedentary when recorded counts were ≤25 counts/15 s. Differences in sedentary time and sedentary fragmentation were examined using the Friedman test. Tracking was examined using Spearman’s correlation coefficients and trajectories over time were assessed using multilevel linear spline modelling.

**Results:**

Median daily sedentary time increased from 51.3 % of waking hours at 7y to 74.2 % at 15y. Sedentary fragmentation decreased from 7y to 15y. The median number of breaks/hour decreased from 8.6 to 4.1 breaks/hour and the median bout duration at 50 % of the cumulative sedentary time increased from 2.4 min to 6.4 min from 7y to 15y. Tracking of sedentary time and sedentary fragmentation was moderate from 7y to 15y however, the rate of change differed with the steepest increases/decreases seen between 9y and 12y.

**Conclusion:**

In this study, sedentary time was high and increased to almost 75 % of waking hours at 15y. Sedentary behavior became substantially less fragmented as children grew older. The largest changes in sedentary time and sedentary fragmentation occurred between 9y to 12y, a period which spans the transition to secondary school. These results can be used to inform future interventions aiming to change sedentary behavior.

## Background

Sedentary behavior (e.g. sitting, screen time) is negatively related to several health outcomes independent of physical activity in adults. More specifically, it has been shown that sedentary behavior, that is sitting time and the fragmentation of sitting time (i.e. the extent to which sitting time is prolonged or interrupted), is related to health outcomes [1, 2]. The evidence on the association between sedentary behavior and health among children and adolescents remains inconclusive [3]. However, sedentary behavior appears to track from childhood into adulthood [4]. Therefore, understanding the change of these behaviors from early life is crucial.

In many parts of the world policy and research interventions to modify sedentary behavior of children and adolescents are now being developed. However, there is still a lack of evidence on which to base such interventions: evidence on basic information is lacking, such as time spent sedentary during childhood and adolescence, the extent to which sedentary time is fragmented, how this behavior changes across childhood and adolescence and how these behaviors interact with light intensity physical activity and/or sleep. Longitudinal evidence on these issues can provide important information for policy makers and researchers designing interventions, and can inform decisions such as when to intervene.

Previous studies reporting on tracking of sedentary behavior have generally focused on only one aspect of sedentary behavior (e.g. television viewing, computer use), were of limited longitudinal duration (i.e. 1, 2 or 3y), did not include the important transition from childhood to adolescence or used self-report methods [5–7]. To our knowledge no evidence is available on the tracking of overall sedentary time, or the fragmentation of sedentary behaviour, from childhood into adolescence. Capturing the transition from childhood into adolescence, in more contemporary cohorts, and including multiple time points throughout childhood and adolescence could highlight crucial periods in life in which changes in sedentary behavior occur and thus inform future timing of interventions to focus on critical time periods. Therefore, the current study aims to broaden the evidence base in this area substantially by 1) providing normative data on sedentary behavior, and longitudinal changes in sedentary behavior, across childhood and adolescence; 2) investigating to what degree sedentary behavior tracks across childhood and adolescence; 3) examine when the greatest changes in sedentary behavior take place.

## Methods

### Participants

Participants were part of the Gateshead Millennium Study cohort. Details of this cohort study have been published previously [8]. Briefly, the GMS is a contemporary cohort which is socio-economically representative (based on the Townsend deprivation index from the UK 1991 census) of North-East England with an equal distribution across all the deprivation quintiles from age 8y [8]. The majority of the participants were from Caucasian backgrounds. The GMS is highly generalizable across the UK in view of the similarity in levels of objectively measured sedentary behavior and in the main determinants (age, gender, season, obesity) of objectively measured sedentary behavior [9]. For the present study, measures collected when children were 6y to 8y of age (October 2006 to December 2007), 8y to 10y (October 2008 to September 2009), 11y to 13y (October 2011 to September 2012) and 14y to 16y were used (September 2014 to September 2015; from here on referred to as age 7y, 9y, 12y and 15y respectively). The study was approved by the Gateshead and South Tyneside Local National Health Service Research Ethics Committee for data collection at 7y and by the Newcastle University Faculty of Medical Sciences Ethics Committee for the 9y, 12y and 15y data collections. Informed written consent was obtained from the parent/guardian of each child, and children provided their assent to participation.

### Objective measurement of sedentary behavior

Sedentary behavior was measured using an ActiGraph GT1M accelerometer (ActiGraph Corporation; Pensacola USA). Accelerometry protocols used in the GMS have been described in detail elsewhere [10, 11]. In brief, participants were asked to wear the accelerometer on the right hip during waking hours for 7 days. Participants recorded the times when the monitor was put on in the morning, taken off at night and any additional periods the monitor had to be removed (e.g. for a bath). Participants were only included if they provided complete wear time diaries. Non-wear time/sleep data were removed manually based on the wear time diaries and visual inspection by a trained researcher before data analyses. It was decided not to define non-wear time using consecutive zeros as this affects the data significantly especially in longitudinal studies where changes in behavioral patterns are very likely [12]. Data were collected in 15-s epochs and included in the analyses if participants had at least three days with 6 h per day of accelerometry data, though in practice the accelerometer wear times were much higher than this (described below) [13]. Epochs were defined as sedentary when recorded counts were ≤25 counts/15 s. This cut point has been widely used to define sedentary time and has shown good agreement with a posture based monitor when measuring sedentary time [14].

### Outcomes

A custom Microsoft Excel macro was used to calculate sedentary time per day and the percentage of sedentary time per day. To assess sedentary fragmentation the average duration of a break and total number of breaks/hour were calculated. It remains unclear how a break in sedentary behavior should be defined [15], but in the current study a break was defined as any period of time ≥1 min of consecutive counts >25 counts/15 s (this would equate to for example 1 min of slow walking). In addition, fragmentation of sedentary behavior was also assessed by calculating the number of sedentary bouts per hour of sedentary time and the number of sedentary bouts lasting 1-4 min, 5-9 min, 10-14 min, 15-29 min and ≥30 min. Last, the duration of bouts making up for 50 % of total sedentary time was calculated. This provides information about how the total sedentary time is fragmented; a shorter bout length indicates total sedentary time is made up out of several short bouts. As previously recommended sedentary bouts were defined as the minimum period of sedentary time without allowing any interruption (i.e. no counts >25 counts/15 s) [15].

### Statistical analyses

Data were tested for normality and found to be skewed. Differences in sedentary time and sedentary fragmentation between time points were examined using the Friedman test. In addition, differences between boys and girls were examined using the Wilcoxon signed rank tests. Differences between changes in sedentary behavior among the least sedentary versus the most sedentary individuals at baseline were assessed using Kruskal-Wallis rank test.

Individual trajectories of change in percentage of time sedentary and fragmentation of sedentary behavior (i.e. bouts per hour of sedentary time) were described using random-effects models with linear splines. Time spent sedentary and sedentary fragmentation were repeatedly measured during four follow-up periods, hence multilevel models with two levels (follow-up period [level 1] within each child [level 2]) were used. These models estimate individual-specific trajectories with no restriction on the number of measures, account for the correlation between repeated measures on the same child and allow for a change in scale and variation over time [16, 17]. Linear splines were used, with knot points at 9y and 12y, to factor in that the changes may not be constant over the full follow-up time period. In this cohort, small but significant seasonal differences in objectively measured sedentary behavior have been observed [9, 18, 19], therefore adjustment for season of measurement was included. The final estimated individual trajectories (for percentage of sedentary time and sedentary fragmentation) were allowed to differ between boys and girls, have a random intercept, allowed to vary with age (random slope over time) and included an indicator variable (as a fixed effect) to account for differing season of measurement. Tracking of sedentary behavior was examined using Spearman’s correlation coefficients. Tracking coefficients <0.30, 0.30-0.60 and >0.60 were classified as low, moderate or good, respectively [20]. All analyses were performed using STATA 12 (StataCorp, College Station, Texas, USA) and trajectories were modelled in MLwiN version 2.33 [21], which was called from Stata version 12 using the runmlwin command.

## Results

### Participant characteristics

Participant characteristics are described in Table 1. At 7y, 9y, 12y and 15y of age a total of 507, 510, 425 and 310 participants provided valid accelerometer measurements, respectively.

**Table 1:** Participant Characteristics and Summary Measures of Sedentary Behavior Variables

Variable	7y *n* = 507 (252 girls, 255 boys)				9y *n* = 510 (265 girls, 245 boys)				12y *n* = 425 (227 girls, 198 boys)				15y *n* = 310 (166 girls, 144 boys)			
	mean	SD	median	IQR	Mean	SD	Median	IQR	mean	SD	median	IQR	mean	SD	median	IQR
Age (years)	7.5	0.5	7.4	7.2, 7.8	9.3	0.4	9.3	9.1, 9.6	12.5	0.3	12.5	12.3, 12.7	15.2	0.4	15.2	14.9, 15.5
Wear time (min/d)	670.7	69.1	680.6	632.4, 716.1	672.9	75.5	679.3	630.7, 725.8	717.8	82.4	729.7	663.8, 782.5	725.5	82.6	738.1	682.3, 785.5
Sedentary time (min/d)	346.5	66.6	349.5	302.6, 383.8	373.1	63.5	373.6	329.1, 417.0	467.3	87.3	466.8	409.4, 524.1	535.4	85.4	542.1	478.7, 595.6
Sedentary time (%/d)	51.6	7.7	51.3	46.4, 56.1	55.4	6.9	55.5	50.6, 60.5	64.9	8.3	64.7	59.8, 70.7	73.5	6.6	74.2	69.3, 78.4
Duration break (min)	3.5	0.3	3.4	3.2, 3.6	3.5^a^	0.3	3.4 ^a^	3.3, 3.7	4.1	3.2	3.7	3.5, 3.9	3.9^b^	0.4	3.8 ^b^	3.6, 4.2
Breaks per hour > 1 min	8.5	1.3	8.6	7.7, 9.4	7.7	1.2	7.7	6.9, 8.5	5.7	1.6	5.8	4.8, 6.7	4.2	1.1	4.1	3.4, 4.9
Bouts per hour of sedentary time	16.7	1.7	16.9	15.9, 17.8	16.7^a^	1.6	16.9 ^a^	15.9, 17.8	15.2	2.3	15.5	13.8, 16.8	12.9	2.5	12.9	11.4, 14.6
Bouts lasting 1–4 min	82.8	13.9	83.6	74.1, 92.5	89.2	14.3	90.0	79.3, 98.7	95.2	17.6	95.4	83.5, 106.3	85.7 ^a^	20.8	85.3 ^a^	73.5, 98.3
Bouts lasting 5–9 min	8.2	2.9	8.2	6, 10.3	10.1	3.3	10.0	8, 12.2	14.2	4.3	14.3	11.3, 17.3	16.6	4.1	16.7	14, 19.3
Bouts lasting 10-14 min	1.7	1.0	1.6	1, 2.3	2.3	1.2	2.0	1.4, 3	3.8	1.8	3.8	2.4, 5.0	5.3	1.9	5.3	4, 6.6
Bouts lasting 15-29 min	0.8	0.7	0.6	0.3, 1.1	1.1	0.9	0.9	0.4, 1.4	2.2	1.5	2.1	1.1, 3.0	3.8	2.0	3.7	2.3, 5
Bouts lasting +30 min	0.3	0.7	0.1	0.0, 0.3	0.2 ^a^	0.3	0.0 ^a^	0, 0.3	0.4	0.7	0.3	0.0, 0.6	1.1	1.0	0.9	0.4, 1.5
Sedentary bout length at 50^th^ percentile of sedentary time (min)	3.4	3.8	2.4	1.9, 3.2	3.0	1.8	2.7	2.2, 3.4	4.6	2.9	3.9	3.1, 5.2	7.3	3.9	6.4	4.8, 8.5

Bouts were defined as minimum period of sedentary time without allowing any interruption; SD, standard deviation; IQR, interquartile range

^a^ not different to 7 years; ^b^ not different to 12 years

### Longitudinal changes in sedentary time

**Fig. 1:**
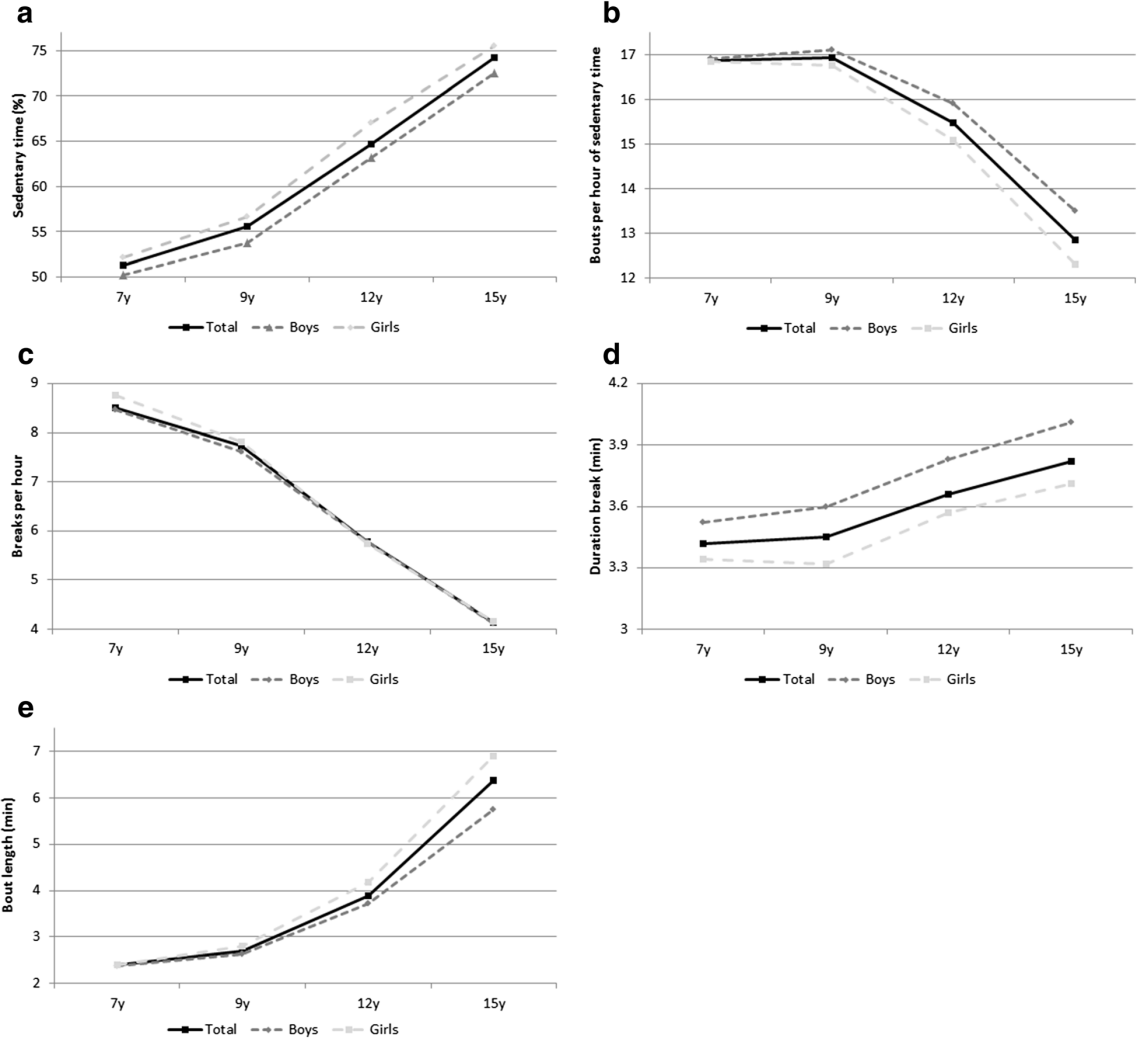
Changes in sedentary behavior from ages 7 to 15 years (median, unadjusted for seasonality). **a**. changes in % sedentary time; **b**. change in sedentary bouts per sedentary hour; **c**. change in breaks/hour; **d**. change in average duration of break; **e**. duration of sedentary bout at 50 % of total sedentary time

**Fig. 2:**
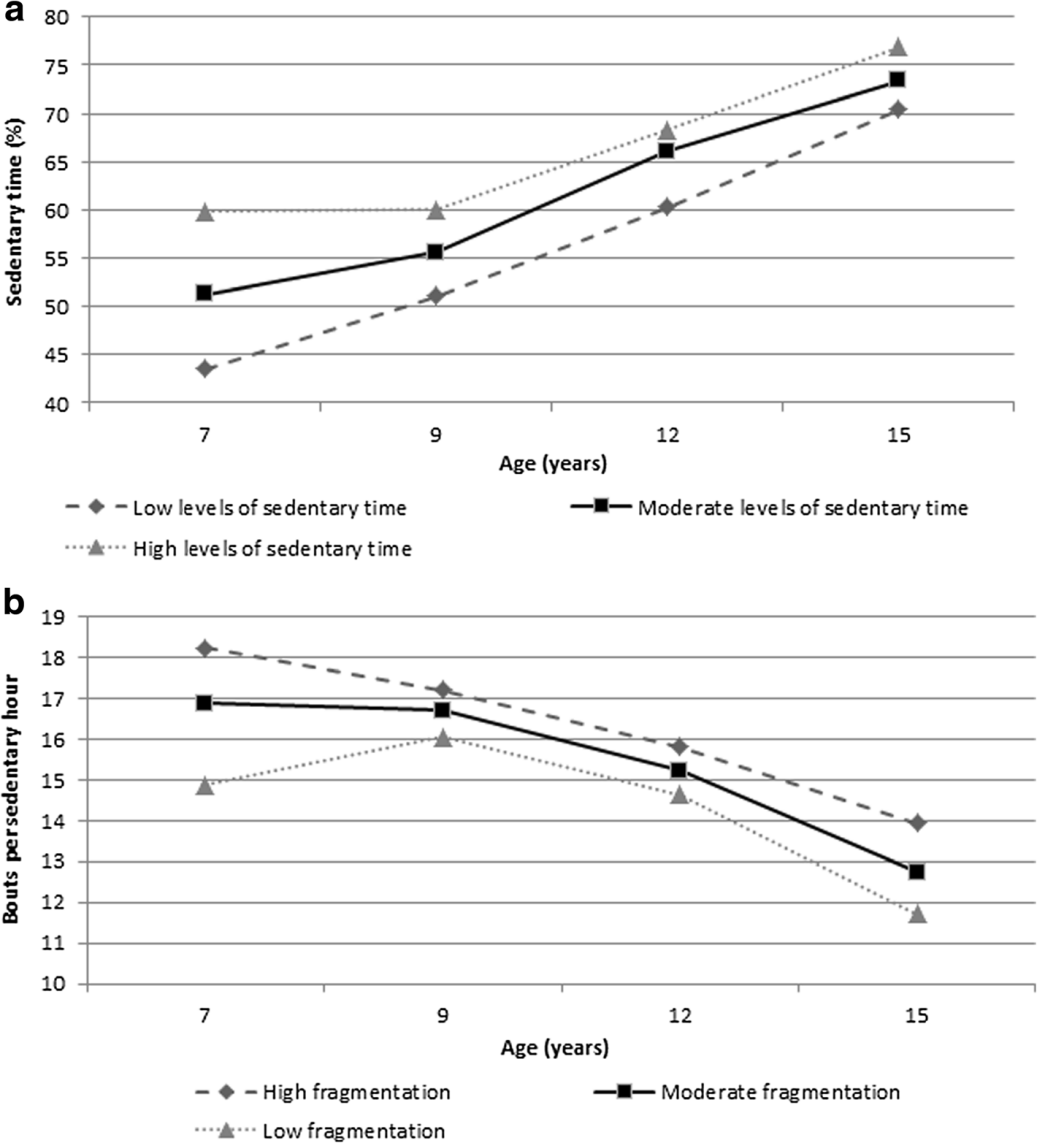
Changes in sedentary behavior from ages 7 to 15 years per tertile (median, unadjusted for seasonality). **a**. sedentary time; **b**. bouts per sedentary hour

Median sedentary time increased every year from 51.3 % (interquartile range 46.4-56.1) per day at baseline (346.5 min/day) to 74.2 % (69.3–78.4) at 15y (535.4 min/day; *p* < 0.05). Changes in median sedentary time are shown in Fig. 1a. Briefly, sedentary time increased by 4.2 % (-0.3-8.6) for 7y to 9y (31.0 min/day), 9.2 % (4.8–13.5) for 9y to 12y (95 min/day), 8.8 % (4.4–12.7) for 12y to 15y (58 min/day). On average sedentary time increased more in girls than in boys (22.8 % versus 22.2 %) however this was not significant (*p* = 0.70). Sedentary time increased more in the least sedentary group compared to the most sedentary group between ages 7y and 9y (6.7 %; 3.3–11.9 versus 0.07 %; -3.6-5.6) and between 12y and 15y (10.3 %; 5.9–15.6 versus 8.0 %; 4.5–11.9; Fig. 2).

**Fig. 3:**
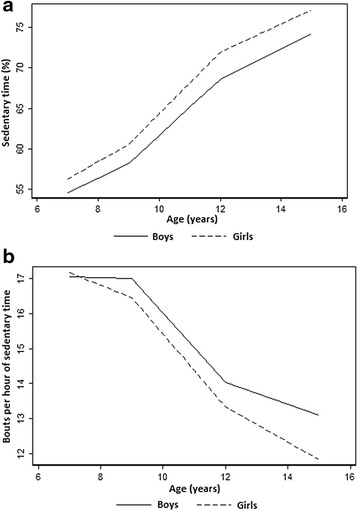
**a**. Average trajectory of percent daily time sedentary over age by sex (adjusted for seasonality). **b**. Average trajectory of sedentary fragmentation (sedentary bouts per hour of sedentary time) over age by sex (adjusted for seasonality)

Trajectory modelling of percentage time sedentary showed non-linear changes as well as sex differences in change over time. For both boys and girls mean percentage sedentary time increased with age adjusting for season of measurement (Fig. 3a). The predicted mean percentage time sedentary at 7y was 47.05 % (SD 4.41) for boys and was 48.62 % (4.18) for girls. Between 7y and 9y the rate of change in mean percentage time sedentary was 1.79 (0.17) for boys and 2.14 (0.17) for girls. Between 9y to 12y there was a steeper rate of increase (3.45; SD 0.17 for boys and 3.78; SD 0.16 for girls), followed by a slower rate of increase between 12y and 15y (1.87; SD 0.17 for boys and 1.74; SD 0.17 for girls).

Pearson’s rank order correlations are shown in Table 2. Sedentary time showed moderate tracking correlations.

**Table 2:** Tracking of daily sedentary behavior from age 7 years to 15 years (Spearman’s coefficient)

Variable	7–9y (*n* = 402)	7–12y (*n* = 326)	7–15y (*n* = 240)	9–12y (*n =* 353)	9–15y (*n* = 263)	12–15y (*n* = 262)
Sedentary time (min )	0.432	0.356	0.277	0.435	0.344	0.459
Sedentary time (%/d)	0.559	0.427	0.428	0.469	0.420	0.503
Duration break (min)	0.565	0.277	0.214	0.288	0.204	0.485
Breaks per hour > 1 min	0.447	0.371	0.362	0.389	0.347	0.488
Bouts per hour of sedentary time	0.310	0.262	0.300	0.385	0.313	0.485
Bouts lasting 1–4 min	0.290	0.159	0.006	0.346	0.117	0.410
Bouts lasting 5–9 min	0.457	0.370	0.089	0.418	0.087	0.313
Bouts lasting 10–14 min	0.443	0.400	0.311	0.399	0.236	0.405
Bouts lasting 15–29 min	0.376	0.304	0.274	0.381	0.325	0.517
Bouts lasting +30 min	0.126	0.033	0.164	0.222	0.061	0.325
Sedentary bout length at 50^th^ percentile of sedentary time (min)	0.414	0.392	0.371	0.411	0.332	0.455

Bouts were defined as minimum period of sedentary time without allowing any interruption

### Longitudinal changes in sedentary fragmentation

Sedentary fragmentation decreased over time. For example, at 7y, the median for breaks per hour was 8.6 (7.7–9.4), whereas this decreased to 4.1 (3.4–4.9) at 15y.

Changes in median fragmentation of sedentary time are shown in Fig. 1b to f. Briefly, the duration of sedentary bouts above which 50 % of sedentary time was accumulated increased from 7y to 15y. In addition, the number of breaks per hour decreased over time as did the number of bouts per hour of sedentary time. On average the number of breaks per hour (-4.4 versus -4.6 breaks/hour for boys and girls, respectively; *p* =0.10) as well as the bouts per sedentary hour (-3.4 versus -4.5 bouts per sedentary hour for boys and girls, respectively; *p* = 0.00) decreased more in girls than in boys.

Trajectory modelling of sedentary fragmentation showed non-linear changes as well as sex differences in change over time (Fig. 3b). The predicted mean fragmentation of sedentary time at 7y was 16.46 (SD 0.41) for boys and was 16.57 (0.39) for girls. Mean fragmentation of sedentary behavior decreased over time for both boys and girls, however, with differing rates of change (Fig. 3b). Between ages 7y and 9y fragmentation decreased at a rate of -0.01 (0.13) for boys and -0.35 for girls (0.13). Between 9y and 12y a faster rate of decline was observed (-0.99; SD 0.13 for boys and -1.43; SD 0.13 for girls), followed by a slower rate of decline between 12y and 15y (-0.31; SD 0.13 for boys and -0.50; SD 0.13 for girls).

Pearson's rank order correlations for tracking sedentary fragmentation are shown in Table 2. Most sedentary fragmentation variables showed moderate correlations.

## Discussion

### Main findings

The current study found high levels of objectively measured sedentary time at age 7y, just over half of the waking day was spent sedentary with increases in sedentary time at age 9y, 12y and 15y so that by 15y typical sedentary time exceeded over 75 % of the waking day (more than US and Canadian adults) [22, 23]. On average, daily sedentary time increased by around 24 min per year. Levels of sedentary time in US and Canadian adults (i.e. 60–70 % of their waking day) in national surveys, measured using the same methods, were similar to those of the GMS cohort participants by age 12y [22, 23]. Sedentary fragmentation also changed significantly and adversely with age, with a decrease in the number of breaks per hour as well as a decrease in bouts per hour of sedentary time from age 7y to 15y. We found medians of 16.9 bouts of sedentary behavior per hour of sedentary time at age 7y and 12.9 at age 15y. This study reported low to moderate tracking coefficients for time spent sedentary and fragmentation of sedentary behavior from childhood into adolescence. However, the gap between participants in the lowest and highest tertiles of sedentary time decreased with age (Fig. 2). In addition, the rate of changes was non-linear and different between boys and girls.

### Comparison with other studies

Since no previous studies have examined changes in objectively measured sedentary time and sedentary fragmentation longitudinally over such an extensive period of childhood and adolescence, and in a contemporary cohort, comparable data are limited. On average, changes in sedentary time and tracking coefficients are similar to those reported previously [4, 6]. A recent study combining data from 20 studies (of which 7 were longitudinal) reported a 20–25 % change in sedentary time between ages 5y–6y and ages 15–16y [24]. These levels are very similar to the results in the current study which show an increase of approximately 22 % of sedentary time between ages 7y and 15y. In addition, the study by Cooper et al. (2015) reported significant differences in sedentary behavior between boys and girls and this was confirmed by the current study [24]. In addition, the rate of change in sedentary behavior differed slightly between boys and girls. In the present study participants’ sedentary time increased on average by 24 min per day per year. This finding is slightly lower than the increase reported in a recent systematic review which reported a weighted average change per year of 30 min/day [6]. The systematic review noted differences between studies, with some studies reporting less change over time. These differences might be due to age group differences, follow up duration as well as methods used to assess sedentary time. For example, the present study has shown that the rate of change in sedentary time appears different during different stages of childhood and adolescence. This means studies examining change from age 7y to 10y might report slightly different increases in sedentary time per year than studies examining change between 12y and 15y of age.

The current study found the largest increase in sedentary behavior happened from age 9y to 12y. This contradicts findings of previous studies using less contemporary birth cohorts which reported a steeper rate of increase in sedentary time in late adolescents compared to early adolescents (i.e. after the transition to secondary school) [25, 26]. A possible explanation for the difference between these studies and the results found in the current study is the decade (i.e. 1990’s versus 2000) in which the cohorts were set up. The current cohort was born 10 years later than cohorts in the previous studies. With the rapid increase in the availability and accessibility of modern technology (e.g. in the UK 57 % of families had internet access in 2006 compared to 86 % in 2015) [27] it may be that children in the GMS would have had access to these modern technologies from a younger age affecting their sedentary behavior pattern earlier on.

Based on the results of this study it is impossible to pinpoint exactly why changes in sedentary time and fragmentation occurred during this period. It may be that the 9–12y period is one in which students become more engaged with electronic media. If this is found to be true it may be important to raise awareness of the negative effects of electronic media use from early on. Also, the 9–12y period spans the transition from primary to secondary education and this transition from one environment (primary school) to another (secondary school) may affect students’ behavior. This may indicate that targeting specific domains (e.g. the secondary school environment) may be needed.

It remains unclear as to which behavior is displaced by the increase in sedentary time with age. In this cohort the increase in sedentary behavior has been larger than the decrease time spent in moderate-to-vigorous intensity physical activity [10] indicating that this behavior is probably not only replacing physical activity but might also impact other behaviors such as light physical activity or sleep. Mitchell et al. (2012) reported the increase in sedentary time was almost equal to the decrease in light intensity physical activity suggesting sedentary time replaces light intensity physical activity [7]. However, a recent meta-analysis (of largely cross-sectional studies with subjective measurement methods) has shown that while sedentary time and total physical activity are inversely associated, the association is weak [28].

This is the first study examining change in sedentary fragmentation and the difference between the rate of change in sedentary behavior of the least sedentary versus the most sedentary children. While sedentary time appears to track from childhood into adolescence (i.e. the most sedentary children remain the most sedentary group as adolescents, Table 2) it is worth noting that the gap between the least sedentary and most sedentary children decreased (Fig. 2). This highlights the need to target all children in order to reduce the age-related changes in sedentary behavior, and not just the most sedentary group. Also, the gap between the most and least sedentary groups decreased most between the ages 7y and 9y and therefore it may be worth targeting sedentary time as early as age 7y.

### Study strengths and limitations

The inclusion of multiple follow-ups, the relatively large sample size, fairly representative sample [8], and the use of objective methods to measure sedentary behavior are strengths of the current study. In addition, thus far no study has reported on the absolute amounts and the degree of tracking of sedentary fragmentation which makes this study very novel.

A number of limitations of the present study should be noted too. There was a fair amount of loss to follow-up. However, there were no differences between BMI, socio-economic status (SES) or sedentary time/fragmentation at baseline between included participants and excluded/lost to follow-up participants (Table 3). No differences were found in change in sedentary time/fragmentation with age between participants who provided valid data for all four data collection points and those who had 1 or 2 data points missing (Table 4), and last the analyses are relatively robust to attrition. In addition, even though SES of the GMS cohort is representative of the SES for families living in the northern parts of England and Scotland, generalisability of our findings to other population groups is not clear and should be established by comparison with future studies. Last, this study focused on overall sedentary behavior and did not examine the difference in change in sedentary behavior during school days and non-school days as this was beyond the scope of the current study. It is therefore not possible to say at what point during the day the biggest changes occur.

**Table 3:** Participants characteristics of follow up versus lost to follow up

Variable	Follow up at age 15y *n* = 240 (130 girls, 110 boys)	Lost to follow up at age 15y *n* = 267 (122 girls, 145 boys)
Height (cm)	124.4	125.3
Weight (kg)	26.09	26.60
BMI (kg/m^2^)	16.71	16.83
SES	2.81	3.01
Sedentary time (%/day)	51.4	51.7
Bouts per hour of sedentary time	16.8	16.5

SES, socio economic status

**Table 4:** Change in sedentary behavior for participants providing data at all 4 time points versus participants with 1 or 2 missing data points (mean, SD)

Variable	7–9y (Mean, SD)		7–12y		7–15y		9–12y		9–15y		12–15y	
	All (*n* 199)	Missing (*n* 203)	All (*n* 199)	Missing (*n* 127)	All (*n* 199)	Missing (*n* 41)	All (*n* 199)	Missing (*n* 154)	All (*n* 199)	Missing (*n* 64)	All (*n* 199)	Missing (*n* 63)
Sedentary time (min)	33.1 (64.4)	28.9 (71.0)	126.1 (85.6)	111.4 (93.8)	191.9 (95.5)	166.8 (81.6)	93.0 (76.9)	93.3 (87.6)	158.8 (85.1)	153.4 (77.6)	65.8 (90.7)	59.5 (90.3)
Sedentary time (%/d)	4.6 (6.3)	3.9 (6.9)	13.6 (8.2)	13.5 (9.1)	22.3 (7.9)	21.4 (6.1)	9.0 (7.7)	9.8 (8.3)	17.7 (7.4)	18.5 (5.2)	8.7 (7.4)	8.7 (7.2)
Bouts per hour of sedentary time	−0.22 (1.6)	0.06 (1.9)	−1.6 (2.4)	−1.4 (2.8)	−4.0 (2.4)	−4.4 (2.6)	−1.3 (2.2)	−1.5 (2.2)	−3.8 (2.3)	−4.0 (2.5)	−2.4 (2.3)	−2.7 (3.0)

*p* > 0.05 for all

## Conclusions

In this sample of English children, sedentary time was high and increased non-linearly from 7y to 15y of age. The largest increase in sedentary time and decrease in sedentary fragmentation was noted from age 9y to 12y, but across all time points changes in sedentary behavior were adverse: the amount of time spent sedentary increased; the fragmentation of sedentary decreased. In adults, it is now well established that high levels of sedentary time and low levels of sedentary fragmentation are associated with increased risk of all-cause mortality, and specifically increased risk of some cancers and cardiometabolic disease [29, 30]. This recent evidence on sedentary behavior and health in adults has led to an increasing emphasis on policy and research interventions to modify sedentary behavior during childhood, and such interventions can be informed by the evidence from the present study. The stability of sedentary time and breaks in sedentary behavior was moderate from age 7y to 15y. This means there is a certain degree of variance in the student’s behavior over this period. This highlights the potential for interventions targeting change in these behaviors. In addition, a larger increase in sedentary time was noted in the children who spent less time sedentary at age 7y. Sedentary behavior became less fragmented as children grew older. The present study suggests that the origin of unhealthy sedentary behaviors in adults may be in childhood and adolescence, and so there is an urgent need for interventions to target sedentary behavior, decreasing overall sedentary time and increasing the fragmentation of sedentary behavior during childhood and into adolescence.

## Abbreviations

IQR, interquartile range; SD, standard deviation; SES, socio-economic status
